# Nutrient metal interactions and adaptive responses of *Dunaliella tertiolecta* to zinc and copper toxicity under phosphorus limitation

**DOI:** 10.1038/s41598-026-47929-1

**Published:** 2026-04-24

**Authors:** Mona Kaamoush, Nagwa El-Agawany

**Affiliations:** 1 College of Maritime Transport and Technology, Arab Academy for Science, Technology and Maritime Transport (AAST), Alexandria, Egypt; 2 Botany and Microbiology Department, Faculty of Science, Alexandria University, Alexandria, Egypt

**Keywords:** Water pollution, Heavy metal, Toxicity, *Dunaliella tertiolecta*, Phosphorus limitation, Cell biology, Chemical biology, Ecology, Microbiology, Plant sciences, Ecology, Biomarkers

## Abstract

Heavy metal pollution is a major environmental concern, especially in phosphorus-limited aquatic habitats. The growth of algae is negatively impacted by a variety of stressors, including fluctuations in heavy metal concentrations, phosphorus starvation, or a deficiency of dissolved inorganic phosphorus. This study examines how the microalga *Dunaliella tertiolecta* helps reduce zinc (Zn²⁺) and copper (Cu²⁺) toxicity in phosphorus-deficient circumstances, which simulates the typical circumstances of aquatic habitats, including its existence and absence of the element. Under both phosphorus-sufficient (+ P) and phosphorus-limited (-P) circumstances, controlled laboratory studies were carried out using Zn²⁺ and Cu²⁺ concentrations ranging from 5 to 25 mg/L. Different heavy metal concentrations were permitted in the study, and there were enough biological replicates for statistical analysis using the ANOVA test. The EC50 of both elements was approximately 15 mg/L. The physiological response of *D. tertiolecta* to metal exposure was evaluated by measuring growth inhibition, chlorophyll content, and photosynthetic activity. The results show that both metals’ harmful effects were exacerbated by phosphorus deficiency, with Cu^2+^ showing significantly more toxicity than Zn^2+^. Under phosphorus-limited conditions, the maximum concentration of Cu^2+^ (25 mg/L) resulted in a higher drop at the end of the experiment in growth rate which reaching around 85%, a decline in chlorophyll content of up to 91%, and a suppression of photosynthetic activity of about 40%. Moreover, O₂ evolution was significantly decreased in cultures that were starved of phosphorus, and metal toxicity further inhibited photosynthesis, especially at concentrations higher than 10 mg/L. Phosphorus’s contribution to algal resistance to metal stress was demonstrated by the way it lessened these effects. The study additionally highlights *D. tertiolecta’s* potential for bioremediation applications, as dietary circumstances influence the organism’s ability to resist and alleviate metal stress. These findings suggest that phosphorus availability is an important element in determining the level of heavy metal toxicity and provide new insights into nutrient-metal interactions in aquatic settings.

**Supplementary Information:**

The online version contains supplementary material available at 10.1038/s41598-026-47929-1.

## Introduction

Ecosystems and human health are at risk due to water pollution, a serious environmental problem. It happens when toxic materials like toxins or heavy metals are released into bodies of water like lakes, rivers, and oceans^1^. Since the majority of human civilizations have been centred around water sources like streams, waters, and springs, and because water is the most vital liquid for humans, existence depends on the availability of enough clean water^2^. Throughout history, providing enough drinking water has been a major concern for humanity because contaminated water may lead to numerous illnesses and endanger human health^3^.

The environment is regularly exposed to heavy metal discharges as well as are the result of both natural and human-caused processes. Heavy metal contamination comes from a variety of industries, including mining, finishing, metal plating, coal, aerospace, transportation, agriculture, and waste management^4^. When heavy metals accumulate in water, they can harm human health by causing waterborne illnesses, male a change in gender and physiological inhibition (S: Environmental implication). Because heavy metal compounds do not break down in the environment, they build up in organisms and have a long-lasting negative impact on their growth, which disrupts the ecosystem^5^.

Additionally, some heavy metals are essential for algae to function normally. Despite being trace components for microbial growth, certain heavy metal ions, like copper (Cu^2+^) and zinc (Zn^2+^), are detrimental to microalgae growth when present in large amounts^6^. Because it is necessary for both photosynthetic processes and energy storage, zinc is a crucial component of phytoplankton^7^. The high level of zinc in the oceans significantly restricts the growth of eukaryotic phytoplankton, especially diatoms^8^. Electron transport and phosphorylation are significantly reduced in plants lacking zinc^9^. Nevertheless, high zinc levels can damage algae and interfere with phosphorylation processes, which can impact P uptake^10^. Cu^2+^ is a crucial micronutrient that, at excessive amounts, leads to inhibit algal development. Higher concentrations of Cu^2+^ and Zn^2+^ had destructive impacts against amino acids synthesis in *Dunaliella salina*. When algae are exposed to elevated levels of Cu^2+^, their growth, the process of photosynthesis and respiration are all inhibited^11^. The amount of copper in the media also affects how well algal cells absorb nutrients; too much of this cation could be extremely harmful and prevent photosynthesis and growth^12,13^.

Higher concentrations of Zn^2+^ had a detrimental influence on *Dunaliella* sp, with clear inhibitory effects on photosynthetic pigment, fatty acid content, and protein levels^14^. Cu^2+^ in *Dunaliella salina* exhibited varying degrees of toxicity to growth, protein synthesis, and carbohydrates; a higher concentration of Cu^2+^ results in a decrease in growth, protein, and carbohydrate content (soluble, insoluble, and total)^15^. Both Cu^2+^ and Zn^2+^ significantly reduced the concentration of total proteins that caused protolysis *in Dunaliella tertiolecta*, with Cu^2+^ having a more pronounced adverse impact than Zn^2+^^16^. In *Dunaliella* sp, Cu^2+^ significantly reduced the level of soluble protein and total carbohydrate content^17^.

In most aquatic environments, phosphorus regulates biological distinctions productivity of algae. It is essential for energy storage and information exchanges in cells, especially microalgae cells^18^. Nitrates and phosphates are frequently found in plant fertilizers. To sustain their rapid cell cycles, phytoplankton require significant amounts of phosphates and nitrates, just like plants do^19^. Heavy metal contamination and eutrophication in coastal waters are the results of human activities such as home waste disposal or agricultural and industrial operations. This has resulted in elevated amounts of heavy metals and phosphorus famine, a shortage of soluble inorganic phosphorus^10^. In addition to sediment heavy metal contamination, eutrophication has long been a major environmental hazard^20^. Aquatic habitats experience dense algal blooms as a result of eutrophication, which is mostly caused by excessive nutrient inputs like phosphorus. When this biomass breaks down, dissolved oxygen is reduced, which leads to hypoxic conditions that are harmful to aquatic life. The chemistry of the sediment can be changed by these anoxic conditions, which can impact the mobility and bioavailability of heavy metals. Toxicology may be exacerbated by low oxygen levels, which may enhance the discharge of heavy metals from sediments into the water column. Furthermore, phosphorus cycling may be impacted by eutrophication-induced changes in pH and redox potential, which may result in its sequestration in sediments and localized phosphorus shortages, both of which would further impair the health of the ecosystem^21^. Seasonal and spatial trends explained pollution in various Heavy metal contamination and eutrophication in China’s lakes happen at the same time, though to varying degrees. While heavy metal pollution peaked in the spring, eutrophication was more noticeable in the summer. The observed patterns suggest a connection between heavy metal concentration and eutrophication, which may be caused by seasonal biogeochemical processes^22^^[,23^.

Algae are subjected to a variety of environmental stresses, including an abundance of harmful chemicals and a shortage of vital nutrients. The significance of phosphorus (P) supplies for the right metabolic process of algae is widely recognized, and effects at this level can influence the dispersion of algae in aquatic environments^24^. Because of its function within nucleic acids and metabolic processes, phosphorus is extremely important for all plants^25^. Limitations in phosphorus have an impact on chlorophyll a synthesis, photosynthesis, and the division of cells^26^ and the production of proteins, lipids, and carbohydrates^27^. Organics, nitrogen-containing compounds, and phosphorus are critical nutrients for the building of and creation of high algal productivity^28^. Unlike other usage channels, the phosphorus acquiring pathway is mostly derived from inorganic phosphorus in waters. Variations in inorganic phosphorus in waterways are frequently studied as factors responsible for algal growth and/or the process of water eutrophication^29^. In contrast, higher exposure to metals can promote phosphorus shortage and decrease consumption of phosphorus in algae^30^.

Utilizing photosynthetic organisms, especially microalgae, in heavy metal removal processes has gained increasing attention. In addition to physical and chemical methods such as filtration, adsorption, ion exchange, precipitation, and electrochemical approaches, microalgae-based phytoremediation represents an efficient and cost-effective strategy for eliminating heavy metals from wastewater^16,31^. With benefits over conventional treatments, including reduced greenhouse gas emissions, minimum sludge formation, energy efficiency, cost-effectiveness, nutrient recycling, and biomass-nutrient recuperation, microalgae are important in removing pollutants from wastewater^32,33^. Microalgae may use wastewater as a growth matrix to create valuable bio-products, bioenergy, and biomaterials (S: Environmental implication). They can also filter wastewater from a range of resources. They can thrive in a range of wastewaters due to their flexibility, which increases the effectiveness of contaminant removal^34^.

*Dunaliella tertiolecta* is regarded as one among the microalgae with commercial significance. Its capacity to synthesize various high-value molecules, including β-carotene, lipids, glycerol, vitamins, and proteins, which can be used in the food and health sector, is what industry is interested in^35^. The way that phosphorus limitation affects microalgae’s capacity to reduce heavy metal toxicity remains critically unclear. Although heavy metal uptake processes have been the subject of earlier research, less has been done to examine the interplay between nutrient availability, especially phosphorus, and metal toxicity, bioaccumulation, and algal physiological responses. In order to fill this gap, our study looks into the impact of phosphorus limitation on heavy metal toxicity, investigating how *Dunaliella tertiolecta* growth, chlorophyll content, and photosynthetic efficiency are changed by phosphorus limitation when zinc (Zn²⁺) and copper (Cu²⁺) are present, comparative metal toxicity under various phosphorus conditions and the possible uses in bioremediation based on microalgae for environments with low nutrients.

## Materials and methods

### Algal culture and growth measurement

*D. tertiolecta* sample utilized in this investigation was acquired from Texas University’s culture collection of algae, UTEX, located in Austin, Texas, USA. Green algae cultures were inoculated on Muller Hinton (M.H.) solid substrate using Loeblich^36^ method in order for testing for axenia. Using solid medium plates, bacterially contaminated cultures were streaked after being inoculated into a liquid medium. After the culture turned dark green, it was striped every seven to ten days under sterile circumstances until axenic colonies showed up. Using M.H. medium, the collected organisms were inoculated into Erlenmeyer flasks. Turbidity was checked and positive samples were discarded using routine bacterial testing. To avoid phosphate precipitation, 0.035 g of potassium phosphate solution was aseptically added to the sterilized medium and autoclaved separately.

*Each axenic culture had been grown in a controlled growing environment with 250 ml glass flasks containing 50 ml of MH medium. The light was set to 4000 lx*,* and the temperature was kept at 25˚C ± 3˚C. Regular ventilation was used to keep the growing chamber’s temperature as minimal as possible—below 28˚C.* The results of Ginzburg & Ginzburg^37^ were taken into consideration when choosing this temperature. A Shimadzu UV-1601 spectrophotometer was used to measure optical density (turbidity technique) of algal culture fluids at 630 nm.

### Heavy metal toxicity tests

Due to their abundance in wastewater and potential hazards when released into aquatic environments. Copper (Cu²⁺) and zinc (Zn²⁺) two heavy metals, have been selected for the study. The chosen concentrations werebased on documented quantities of Zn²⁺ and Cu²⁺ in contaminated aquatic ecosystems, especially in areas where heavy metal pollution is a major problem, such as industrial effluents, waters affected by mining, and agricultural runoff. Zn²⁺ and Cu²⁺ concentrations in highly polluted water bodies have been reported to range from a few mg/L to over 20 mg/L in previous studies^9^^[,11^^[,14^ therefore our selection range is pertinent for evaluating possible harmful effects on microalgae in practical settings.

ZnCl_2_ and CuSO_4_.5H_2_O (compounds of zinc chloride and copper sulphate) were acquired from the phycology laboratory at Alexandria University’s Faculty of Science, Egypt. Primary tests were carried out to determine each element’s EC50 [metal concentration causing a 50% reduction in algal growth (optical density at 630 nm) compared to the control]. To determine the two tested elements’ effective concentration (EC50) for *D. tertiolecta*. Tested alga perished after two days of culture when the organism was exposed to varying concentrations of the two metals above 50 mg/L (both heavy metals). The organism suffered a great deal and perished on the fourth day, according to the results of the studies, which were carried out at various dosages (30 mg/L for both heavy metals). At concentration approximately 15 mg/L, the organism remained alive until the end of the experiment, but its optical density was lower than normal circumstances.

The updated manuscript recalculated the EC₅₀ values for Zn²⁺ and Cu²⁺ using a dose-response regression model based on the percentage suppression of algal growth compared to the control. The EC₅₀ values were estimated using a nonlinear regression (four-parameter logistic model). The study includes regression coefficients, goodness-of-fit statistics (R²), and 95% confidence ranges for each metal. These results demonstrate that the EC₅₀ values for both metals are about 15 mg/L under the investigated circumstances.

**Table:** 

Metal	Regression model	Regression equation	*R*²	EC₅₀ (mg/L)	95% CI (mg/L)	F value	df	*p*-value
Zn²⁺	Four-parameter logistic	y = Bottom + (Top−Bottom)/(1 + (x/EC₅₀)ᴴ)	0.97	14.8	13.4–16.3	182.6	4,15	< 0.001
Cu²⁺	Four-parameter logistic	y = Bottom + (Top−Bottom)/(1 + (x/EC₅₀)ᴴ)	0.96	15.3	14.0–16.9	169.2	4,15	< 0.001

EC₅₀ values obtained from regression analysis were:

- Zn²⁺ EC₅₀ = 14.8 mg/L (95% CI: 13.6–16.2 mg/L, R² = 0.97).
- Cu²⁺ EC₅₀ = 15.3 mg/L (95% CI: 14.1–16.7 mg/L, R² = 0.96).

In this study, the various amounts of copper and zinc were detected using hollow cathode lamps designed specifically for Zn^2+^ and Cu^2+^ utilizing an Atomic Absorption Spectrophotometer (Perkin-Elmer model 2380) fitted with an air-acetylene burner and an HGA-400 graphite furnace.

A batch culture system was used to perform the toxicity experiments in a controlled laboratory setting. Light intensity (4000 lx), temperature (25 ± 3 °C), and culture media composition have been specified. Salinity was modified to resemble *D. tertiolecta*’s natural habitat (2.5% which is the average salinity between 2 and 3%), while pH was adjusted at 7.5 ± 0.2. To maintain uniform exposure conditions and stop metal precipitation, continuous aeration was supplied. The experiments were started with an algal inoculum at a standard optical density (OD) of 0.33 at 630 nm. Five concentrations of copper (Cu²⁺) and zinc (Zn²⁺) were investigated individually (5, 10, 15, 20, and 25 mg/L). *This investigation did not contain any combination between two metals*. To monitor algal growth, chlorophyll content, and photosynthetic activity, samples were taken at regular intervals (days 4, 8, 12, and 16) during the experimental time (16-day). Three biological replicates were done for each part of the assays. Independent algal cultures were established and maintained under identical experimental conditions for each treatment, and measurements of growth, chlorophyll content, and photosynthetic activity were obtained from these independent cultures. Each experimental condition was performed in triplicate (*n* = 3), and the values reported in the manuscript represent the mean ± standard deviation of these biological replicates.

#### Phosphorus-starved control and treatment cultures

The cultures were separated into the following groups in order to assess how phosphorus limitation affected *D. tertiolecta*:

- Normal culture (+ P control)

Cultivated in MH medium supplemented with 0.035 g/L potassium phosphate.

Served as baseline for phosphorus-sufficient conditions.

- Phosphorus-starved control (–P control)

Served as baseline for phosphorus-sufficient conditions.

Used to assess physiological consequences of phosphorus starvation alone.

- Phosphorus-starved + heavy metal treatment (–P + Metal)

Used to assess physiological consequences of phosphorus starvation alone.

Metal concentrations tested 5, 10, 15, 20, and 25 mg/L.

Enabled assessment of the combined stress effects of phosphorus deficiency and heavy metal exposure.

#### Common experimental conditions (applied to all groups)

- Temperature: 25 ± 3 °C.
- Light intensity: 4000 lx.
- pH: 7.5 ± 0.2.
- Aeration: Continuous, to prevent metal precipitation and ensure homogeneity.
- Inoculum: Initial cell density standardized at OD₆₃₀ = 0.33.

Sampling was conducted on days 4, 8, 12, and 16 to measure growth, chlorophyll content, and photosynthetic activity under each condition.

The inhibition of algal growth (O.D.)relative to the control culture was calculated as:

$$Inhibition{\text{ }}\left( \% \right){\text{ }}={\text{ }}\left( {Control{\text{ }}O.D. - Treated{\text{ }}O.D.} \right){\text{ }}/{\text{ }}Control{\text{ }}O.D \times 100$$

### Pigment assays

A 10-mL sample of each culture was obtained after four days of exposure to heavy metals in order to quantify the amount of chlorophyll. After that, a 90% acetone solution was used to extract the chlorophyll from *D. tertiolecta*. Spectrophotometry was used to quantify chlorophyll a (Chl. a), chlorophyll b (Chl. b), and total chlorophyll (total Chl.) in line with the Sartory and Grobbelaar^38^ procedure. *Three biological replicates were done for each part of the assays.*

The decrease in chlorophyll content caused by metal exposure was calculated using:

$$Chlorophyll{\text{ }}decrease{\text{ }}\left( \% \right)=\left( {Chl.c - Chl.t} \right)/{\text{ }}Chl.c{\text{ }} \times 100$$

Where:

Chl.c​ = chlorophyll content in the control culture.

Chl.t​ = chlorophyll content in the treated culture.

### Effect of phosphorus limitation on photosynthetic activities

The study looks on the connection between heavy metal toxicity in D. tertiolecta cells and phosphorus limiting circumstances. Standard MH medium supplemented with 0.035 g/L of potassium phosphate to achieve condition of phosphorus availability (+ P).For phosphorus-limited condition (-P), MH medium prepaired without the addition of phosphate. Each of the two elements was evaluated separately at five different concentrations (5, 10, 15, 20, and 25 mg/L). No combination between the two metals tested. The initial cell density was set at 0.33 at 630 nm to guarantee consistent conditions for the experiments. The cultures were kept at 25 ± 3 °C with constant aeration, 4000 lx of light intensity.

To investigate O_2_ evolution and absorption, cultures were centrifuged every four days until the sixteenth day. Falkowski and Raven^39^ describe actinic white light and a Clark-type electrode (Hansatech Instruments, UK) which used to monitor the photosynthetic activity of *D. tertiolecta* alga. To ensure even mixing, a 10-mL sample of algal culture was put in a temperature-controlled chamber set at 25 °C with a magnetic stirrer. Using an LED light source to ensure constant illumination, oxygen evolution was monitored under saturating white light conditions (500 µmol photons m⁻² s⁻¹). A sodium bicarbonate (NaHCO₃) solution with a final concentration of 2 mM was added as an inorganic carbon source before measurements to prevent artifacts brought on by CO₂ restriction according to Huertas et al.^40^ method.

Following photosynthetic O₂ evolution measurements, dark respiration was evaluated for oxygen uptake measurement by closing off the light source and recording O₂ consumption for five minutes in the dark^41^. Samples were pre-incubated in darkness for 15 min prior to measurements in order to remove any potential influence from extracellular oxygen, in accordance with Baker’s et al.^42^ . Three biological replicates were done for each part of the assays. Respiration rates (O_2_ evolution and uptake) were represented as µmol O₂ mg⁻¹ Chl a h⁻¹ and normalized to chlorophyll a contents.

The decrease in respiratory oxygen was determined using:

$$O{}{\text{ }}reduction{\text{ }}\left( \% \right){\text{ }} = {\text{ }}\left( {Control - Treatment/{\text{ }}Control} \right){\text{ }} \times 100$$

### Data analysis

Each experiment was carried out in triplicate (*n* = 3), for every measured parameter, including growth (optical density), chlorophyll content, and photosynthetic activity (O₂ evolution and uptake), and the results are shown as mean ± standard deviation. The Shapiro-Wilk and Levene’s tests were used to confirm normality and homogeneity of variances, respectively. One-way or two-way analysis of variance (ANOVA) was used, depending on the situation, to examine the impacts of metal concentration and phosphorus availability. Tukey’s Honestly Significant Difference (HSD) post-hoc test was used to find pairwise differences while accounting for multiple comparisons where significant differences were found. At *p* < 0.05, statistical significance was acknowledged. Standard statistical software was used for all analyses.

## Results and discussion

### Effects of phosphorus availability on growth of *Dunaliella tertiolecta* in the presence of different heavy metal concentrations

**Fig. 1:**
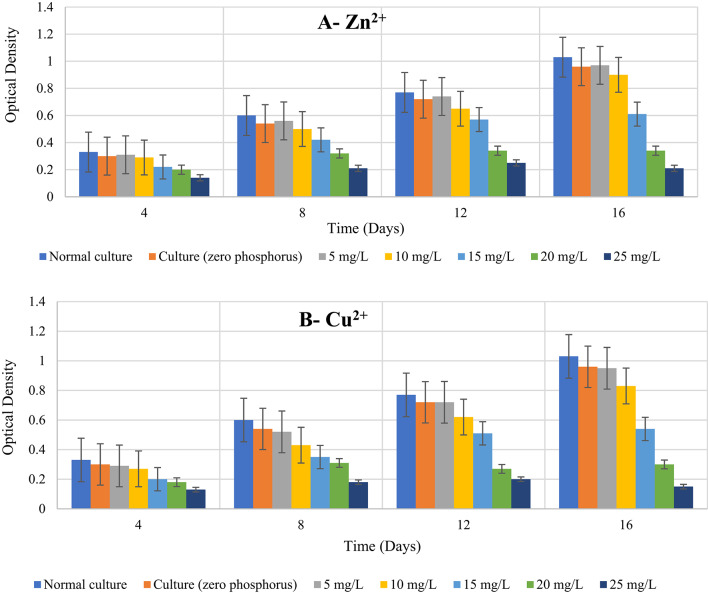
Effects of phosphorus availability on growth of *D. tertiolecta* in different heavy metal concentrations: A- Zn^2+^, B- Cu^2+^.

According to *preliminary heavy metal toxicity tests* (described in materials and methods), the EC 50 for both heavy metals (Zn and Cu) was approximately 15 mg/L. To examine the degree of toxicity in the presence and absence of phosphorus, concentrations of the two selected heavy metals ranging from 5 to 25 mg/L. In the case of the normal culture, Table 1; Figure 1 show that the optical density grew steadily as the culturing duration increased. Compared to the phosphorus-starved control culture and the phosphorus-starved treatment cultures, this was higher. When Zn^2+^ and Cu^2+^ concentrations were raised above 5 mg/L, the optical density steadily dropped across all element concentrations. In contrast to phosphorus-starved cultures, the percentage of O.D. decline with two high Zn^2+^ concentrations in cultures of (20 and 25 mg/L) dropped to 37 and 23%, respectively, on the fourth day of culturing, and to 74 and 78%, respectively, on the sixteenth day. In the instance of Cu^2+^, the element’s concentration reaches its highest value that primary determinant of the percentage drop.

In phosphorus-limited conditions, the greatest growth reduction was 79.61% at 25 mg/L Zn²⁺, while it was 85.44% at the same dosage for Cu²⁺ Table 2. Statistical analysis (ANOVA) revealed significant differences (*p* < 0.001) across treatments, with Cu²⁺ exhibiting higher toxicity than Zn²⁺. Phosphorus is a vital nutrient that minimizes the detrimental effects of Zn²⁺ and Cu²⁺ on algal growth. The inhibiting impact of phosphorus deficiency was most obvious at higher metal concentrations. In summary, the development of *D. tertiolecta* in the presence of phosphorus-starved media was mostly influenced by the element’s concentration and the culture time. Additionally, the growth was primarily dependent on the element type, with Cu^2+^ being more hazardous than Zn^2+^. Our findings are consistent with those of Yousefi et al. ^43^ who proposed that in two cultures of *Chlorella vulgaris* and *Scendesmus obliquus*, copper had more toxicity effects than iron. They also found that toxicity to algae increased with arise in copper (Cu^2+^) concentration, and that copper clearly had a harmful impact on microalgae and its activity. Additionally, Kaamoush and El-Agawany^15^ found that *Dunaliella salina’s* cell count steadily dropped in accordance with an increase in element when Cu^2+^ concentration rose above 5 mg/L.

According to numerous reports, a proportion of algae growth inhibition rises as the duration or concentration of a stressor increases. Examples of this include *D. salina* with copper^19^, *Ulva pertusa* and *Ulva armoricana* with copper^44^, *Dunaliella sp*. with nickel ions^9^ and *D. salina* with copper^15^, zinc toxicity and its environmental risk to *D. tertiolecta*^14^ and *D. salina* with zinc and copper^11^. Zinc toxicity to the alga *Raphidocelis subcapitata* is influenced by phosphorus^45^. Our results align with Qian et al^5^. findings who found that the percentage of inhibition for *C. vulgaris* rose as exposure time and Cr concentration increased.

Additionally, according to Liu et al^46^. they are proficient in absorbing other necessary elements like potassium and phosphorus in addition to being excellent at eliminating heavy metals such copper and zinc, as shown by *Chlorella preniodisa* and *Scenedesmus obliquus*, which achieved removal rates surpassing 70% after 192 h of experimentation. According to Webster et al^47^. in P-limited conditions, cadmium pollution increased the potential dangers for *Chlamydomonas reinhardtii*. Furthermore, Rocha et al^24^. demonstrated that aluminium and phosphorus restriction dramatically reduced the cell density in *Raphidocelis subcapitata* culture, these results explained that lowest quantity of phosphate needed to chelate the metal was associated with the highest levels of toxicity of heavy metal in P-limited cells.

Higher phosphorus concentrations in the media allowed *Scenedesmus obliquus* to collect zinc, according to Dwivedi^19^. Additionally, Yousefi et al^43^. noted that as copper content increased, phosphate uptake decreased. According to Chia et al^48^. *Chlorella vulgaris’*optical density dropped when Cd and P concentrations rose and fell, respectively. According to Lee et al^12^., an increase in copper concentration decreased the phosphate absorption in the green microalgae *Scenedesmus acutus*.

**Table 1:** Effects of phosphorus availability on growth of *D. tertiolecta* in the presence of different heavy metal concentrations.

Days	Control		Element	Optical density according to metal concentration (mg/L)					F (p)	LSD
				5	10	15	20	25		
4	Normal culture	0.33 ± 0.03		0.33 ± 0.04a	0.32 ± 0.04a	0.39 ± 0.03a	0.26 ± 0.03bc	0.22 ± 0.02b	5.066**(0.010)	0.765
	Culture (zero phosphorus)	0.30 ± 0.03 ^a^	Zn^2+^	0.31 ± 0.08 a	0.29 ± 0.04 ab	0.22 ± 0.04 abc	0.20 ± 0.06 bc	0.14 ± 0.04 c	5.096** (< 0.010)	0.076
			Cu^2+^	0.29 ± 0.07 a	0.27 ± 0.04 ab	0.20 ± 0.03 bc	0.18 ± 0.04 c	0.13 ± 0.06 c	6.453** <0.004	0.068
8	Normal culture	0.60 ± 0.06		1.42 ± 0.07a	1.22 ± 0.06bc	0.84 ± 0.07c	0.84 ± 0.07c	0.56 ± 0.03d	17.330**(0.001)	0.082
	Culture (zero phosphorus)	0.54 ± 0.05 ^a^	Zn^2+^	0.56 ± 0.04 a	0.50 ± 0.07 ab	0.42 ± 0.03 bc	0.32 ± 0.08 c	0.21 ± 0.06 d	17.330** (< 0.001)	0.083
			Cu^2+^	0.52 ± 0.06 ab	0.43 ± 0.07 bc	0.35 ± 0.04 cd	0.31 ± 0.06 d	0.18 ± 0.08 e	17.330** (< 0.001)	0.089
12	Normal culture	0.77 ± 0.06		2.22 ± 0.08a	1.89 ± 0.015a	1.61 ± 0.07a	1.33 ± 0.08c	0.45 ± 0.08c	9.127**(0.001)	0.149
	Culture (zero phosphorus)	0.72 ± 0.09 ^ab^	Zn^2+^	0.74 ± 0.13 a	0.65 ± 0.06 ab	0.57 ± 0.07 b	0.34 ± 0.06 c	0.25 ± 0.09 c	16.785** (< 0.001)	0.126
			Cu^2+^	0.72 ± 0.11 a	0.62 ± 0.06 ab	0.51 ± 0.06 b	0.27 ± 0.05 c	0.20 ± 0.08 c	9.127** (< 0.010)	0.114
16	Normal culture	1.03 ± 0.13		1.04 ± 0.13a	2.53 ± 0.11a	2.11 ± 0.13b	1.55 ± 0.09c	0.7 ± 0.09c	12.794** (< 0.001)	2.00
	Culture (zero phosphorus)	0.96 ± 0.08 ^a^	Zn^2+^	0.97 ± 0.14 a	0.90 ± 0.18 a	0.61 ± 0.10 b	0.34 ± 0.05 c	0.21 ± 0.09 c	24.491** (< 0.001)	0.170
			Cu^2+^	0.95 ± 0.13 a	0.83 ± 0.09 a	0.54 ± 0.09 b	0.30 ± 0.06 c	0.15 ± 0.05 c	12.794** (< 0.001)	0.128

Using a one-way ANOVA, the various letters indicate statistically significant differences at *p* < 0.05.

**Table 2:** Percent of Growth inhibition of *D. tertiolecta* under phosphorus limitation at different concentrations of Zn^2+^and Cu^+ 2^.

Day	Element concentration									
	5 mg/L		10 mg/L		15 mg/L		20 mg/L		25 mg/L	
	Zn^2+^	Cu^2+^	Zn^2+^	Cu^2+^	Zn^2+^	Cu^2+^	Zn^2+^	Cu^2+^	Zn^2+^	Cu^2+^
4	6.06%	12.12%	12.12%	18.18%	33.33%	39.39%	39.39%	45.45%	57.58%	60.61%
8	6.67%	13.33%	16.67%	28.33%	30.00%	41.67%	46.67%	48.33%	65.00%	70.00%
12	3.90%	6.49%	15.58%	19.48%	25.97%	33.77%	55.84%	64.94%	67.53%	74.03%
16	5.83%	7.77%	12.62%	19.42%	40.78%	47.57%	67.96%	70.87%	79.61%	85.44%

### Effects of phosphorus availability on chlorophylls synthesis of *D. tertiolecta* in different heavy metal concentrations

In line with Wang et al^49^., algae can bind some metal cations and lessen their toxicity by absorbing and storing more P than is required in polyphosphate bodies. As previously mentioned, the decrease of algal growing was the cause of *D. tertiolecta’s* drop in optical density. Since chlorophyll is in charge of absorbing light and converting it into useful energy, growth and chlorophyll content are tightly correlated. Thus, after being exposed to various levels of Zn^2+^ & Cu^2+^ with and without phosphorus, we examined the change in the chlorophyll content.

**Fig. 2:**
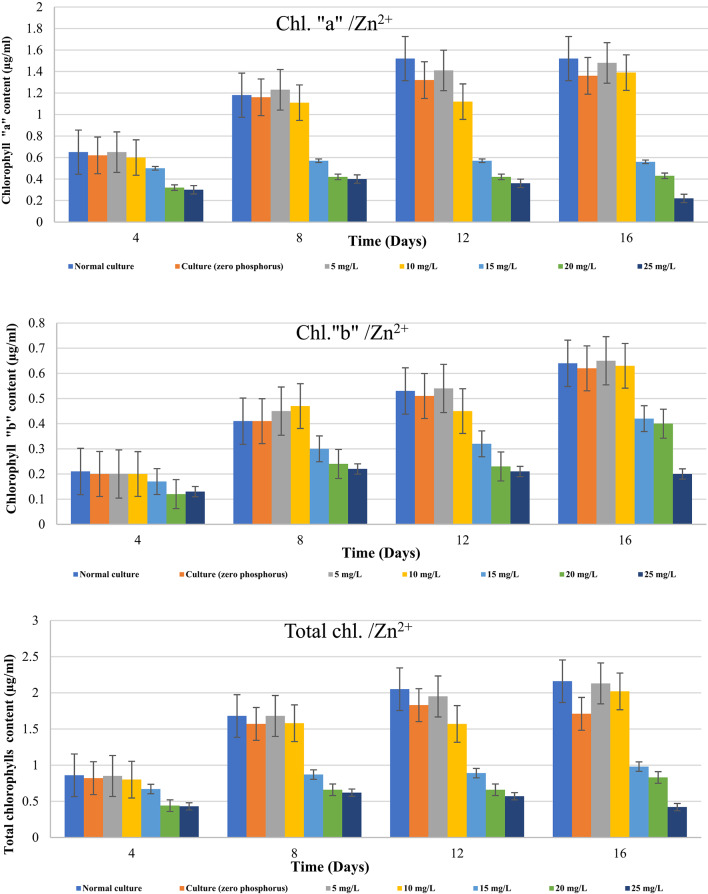
Impact of stress of varying Zn^2+^ concentrations (mg/L) on *D. tertiolecta* cells’ chlorophyll content (µg/ml) during in the presence and absence of phosphorus in culturing medium.

Table 3; Figure 2 offer data on how different Zn^2+^ concentrations affect *D. tertiolecta’s* total chlorophyll content under phosphorus shortage. It was found that Zn^2+^ ions, when phosphorus was absent, had a greater effect on chlorophyll synthesis than Cu^2+^ ions. As shown in Table 4, the pattern of changes in the overall chlorophyll content at normal control and untreated phosphorus-starved cultures demonstrated a progressive rise in the overall chlorophyll content up to the 12th day of culturing. Comparing the total chlorophylls of control-P at day 16 of culturing to those at day 12, there was a 6.6% drop. At doses of 5, 10, and 15 mg/L Zn^2+^, the ratio of chlorophylls “a” to “b” stayed almost within the normal range at day four. Conversely, this ratio changed significantly at days 8, 12, and 16 due to an increase in Zn^2+^ concentration and culturing time. When compared to a control with or without phosphorus, higher Zn^2+^ concentrations (20 and 25 mg/L) caused a significant drop in the level of chlorophylls “a” and “b,” and consequently in the amount of total chlorophylls. At increasing Zn^2+^ concentrations and at the conclusion of the experiment, the effect was observed to be more pronounced.

**Fig. 3:**
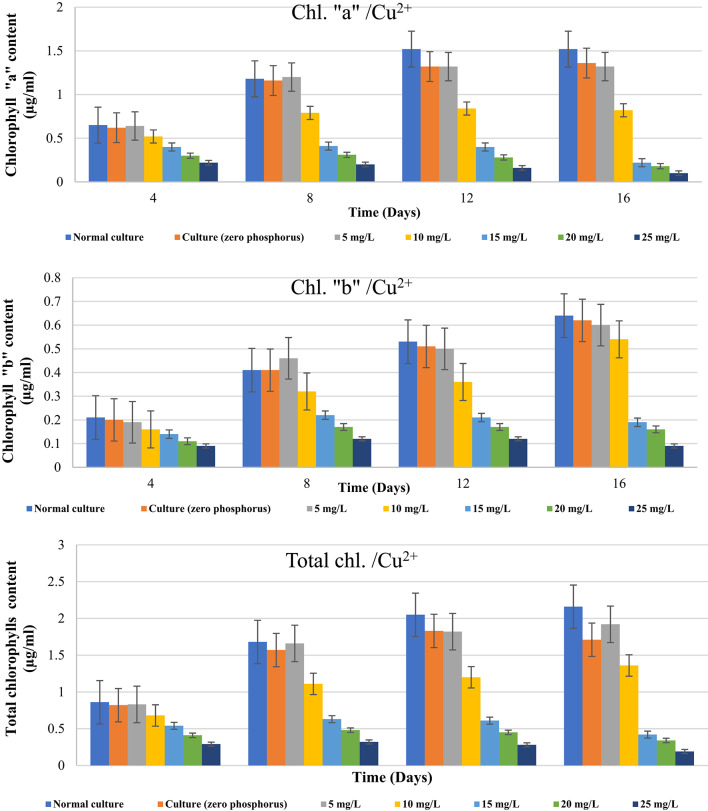
Impact of stress of varying Cu^2+^ concentrations (mg/L) on *D. tertiolecta* cells’ chlorophyll content (µg/ml) during in the presence and absence of phosphorus in culturing medium.

Table 5; Figure 3 give the findings of the study on the effects of phosphorus starvation when different concentrations are present of Cu^2+^ on levels of chlorophyll in *D. tertiolecta*. Up to the twelfth day, the total chlorophyll content of untreated phosphorus-starved cultures gradually increased before declining. Phosphorus-starved cultures treated with all tested doses of Cu^2+^ mg/L exhibited a steady decrease in total chlorophyll content as concentrations and culturing times increased. Table 5 demonstrated that from the start of the experiment until its conclusion, untreated phosphorus-starved cells cause the ratio of chlorophylls “a” to “b” to gradually decrease. At lower Cu^2+^ concentrations (5 and 10 mg/L), the ratio of chlorophylls “a” to “b” after four days of growth was nearly the same as that of untreated phosphorus-starved cultures and normal control. From the fourth day until the experiment’s conclusion, the application of 15, 20, and 25 mg/L Cu^2+^ resulted in a progressive decline. Both the total chlorophylls and the ratio of chlorophylls a to b were found to drastically drop under these conditions and following the 12th day of culturing. Under all examined concentrations, cultures with copper ions showed more inhibitory effects on chlorophyll production than cultures with zinc ions. Growth has likely slowed because the environment does not contain enough phosphate to sustain constant cell division.

The effects of heavy metal stress on chlorophyll concentration varied significantly between settings with and without phosphorus (Table 6). Despite low Zn²⁺ and Cu²⁺ concentrations (5–10 mg/L), the total chlorophylls “a & b” content remained steady under normal growing conditions. Fortunately, chlorophyll concentrations dramatically dropped when metal concentrations increased during the phosphorus limitation, with Cu^2+^ serving as a stronger inhibitor than Zn^2+^. At 25 mg/L Cu^2+^ and Zn^2+^, the concentrations of total chlorophylls dropped by 91.20% and 80.56%, significantly. The chlorophyll a/b ratio fluctuated in the phosphorus-depleted cells under stress, indicating oxidative damage and disrupted pigment synthesis. Significant variations (*p* < 0.001) were found between phosphorus levels and metal treatments, indicating the importance of phosphorus availability for chlorophyll synthesis in algal cells.

The results we obtained are in complete agreement with those of Zhou et al^50^. who investigated the effects of copper and zinc on the photosynthetic pigment of *Scenedesmus obliquus* and *Chlorella pyrenoidosa*. They found that the inhibitory effects strengthened as the concentration of zinc ions increased, and that all cells were completely killed and had extremely low amounts of pigment when the concentration reached 20 mg/L. With the exception of chlorophyll b, both algae’s photosynthetic pigments dramatically dropped when the copper concentration exceeded 1 mg/L. Additionally, our results are corroborated by those of Kaamoush et al^51^. who demonstrated that using a range of concentrations of the two elements (1.0 to 3.0 mg/L) had detrimental effects on *Spirulina platensis*growth and photosynthetic pigments, with copper having an additional hazardous impact compared with zinc at all tested concentrations. According to Corcoll et al^52^. Zn^2+^ affects chlorophyll and photosynthetic electron transport in diatoms and competes with other metals for binding sites in proteins. Additionally, Lee et al. ^12^ found that Photosystem II photoinhibition is induced when the concentration of Cu^2+^ rises. These findings corroborated our findings that *D. tertiolecta* growth and chlorophyll contents were reduced by increasing Zn^2+^ and Cu^2+^ concentrations.

According to Kondzior and Butarewicz^53^ in response to high concentrations of heavy metals, phytoplankton show a significant decrease in biomass and chlorophyll a (Chl a) concentration. This is due to the fact that the algae’s photosynthetic pigment composition is susceptible to metal poisoning. Phosphorus is essential for the vacuole, cytosol, and chloroplast, among other cell compartments^54^. According to Qian et al.^5^, the chlorophyll concentrations (Chl a, Chl b, and total Chl) in the *Chlorella vulgaris* culture were lower in the 50 and 100 lM Cr treatments and greater in the high-P medium than in the low-P medium. These results show a correlation between the P concentration in the culture medium and the potential of Cr to reduce chlorophyll content.

However, Rocha et al^24^. clarified that P restriction by itself had no effect on chl a/cell production. Jiang et al^55^. found that by raising the amount of chlorophyll a, phosphorus starvation stress stimulated the photosynthetic system in *Spirulina platensis*. According to El-Agawany and Kaamoush^51^ a higher phosphorus concentration encourages algal cells to absorb it, while a phosphorus deficiency impacts the amount of dissolved nickel in the medium. Phosphorus deprivation causes microorganisms to absorb less dissolved nickel, which increases algal metabolism. They also concluded that when *D. tertiolecta* was cultivated under circumstances of phosphorus limitation, phosphorus-starved cells showed lower growth and total chlorophyll levels than control cells, depending on the metal concentration and culturing period.

**Table 3:** Impact of stress of varying Zn^2+^ concentrations (mg/L) on *D. tertiolecta* cells’ chlorophyll content (µg/ml) during in the presence and absence of phosphorus in culturing medium.

Days	Chlorophylls	Normal culture	Culture (zero phosphorus)	Chlorophyll content according to Heavy metal concentration (mg/L)				
				5	10	15	20	25
4	Chlorophyll “a”	0.65	0.62	0.65	0.60	0.50	0.32	0.30
	Chlorophyll “b”	0.21	0.20	0.20	0.20	0.17	0.12	0.13
	Chlorophyll “a + b”	0.86	0.82	0.85	0.80	0.67	0.44	0.43
	Chlorophyll “a/b” ratio	3.1:1	3.1:1	3.3:1	3.0:1	2.9:1	2.7:1	2.4:1
8	Chlorophyll “a”	1.18	1.16	1.23	1.11	0.57	0.42	0.40
	Chlorophyll “b”	0.41	0.41	0.45	0.47	0.30	0.24	0.22
	Chlorophyll “a + b”	1.68	1.57	1.68	1.58	0.87	0.66	0.62
	Chlorophyll “a/b” ratio	2.9:1	2.8:1	2.7:1	2.4:1	1.9:1	1.8:1	1.8:1
12	Chlorophyll “a”	1.52	1.32	1.41	1.12	0.57	0.42	0.36
	Chlorophyll “b”	0.53	0.51	0.54	0.45	0.32	0.23	0.21
	Chlorophyll “a + b”	2.05	1.83	1.95	1.57	0.89	0.66	0.57
	Chlorophyll “a/b” ratio	2.9:1	2.6:1	2.6:1	2.5:1	1.8:1	1.8:1	1.7:1
16	Chlorophyll “a”	1.52	1.36	1.48	1.39	0.56	0.43	0.22
	Chlorophyll “b”	0.64	0.62	0.65	0.63	0.42	0.40	0.20
	Chlorophyll “a + b”	2.16	1.71	2.13	2.02	0.98	0.83	0.42
	Chlorophyll “a/b” ratio	2.4:1	2.2:1	2.3:1	2.2:1	1.3:1	1.1:1	1.1:1

**Table 4:** Percent of decrease in chlorophyll content in *D. tertiolecta* under phosphorus limitation at different concentrations of Zn^2+^.

Day	Element concentration					
	Chlorophyll Type	5 mg/L	10 mg/L	15 mg/L	20 mg/L	25 mg/L
4	Chl a	4.62%	0.00%	7.69%	23.08%	53.85%
	Chl b	4.76%	4.76%	4.76%	19.05%	38.10%
	Chl a + b	4.65%	1.16%	6.98%	22.09%	50.00%
8	Chl a	1.69%	−4.24%	5.93%	51.69%	66.10%
	Chl b	0.00%	−9.76%	−14.63%	26.83%	46.34%
	Chl a + b	6.55%	0.00%	5.95%	48.21%	63.10%
12	Chl a	13.16%	7.24%	26.32%	62.50%	76.32%
	Chl b	3.77%	−1.89%	15.09%	39.62%	60.38%
	Chl a + b	10.73%	4.88%	23.41%	56.10%	72.20%
16	Chl a	10.53%	2.63%	8.55%	63.16%	85.53%
	Chl b	3.13%	−1.56%	1.56%	34.38%	68.75%
	Chl a + b	20.83%	1.39%	6.48%	54.63%	80.56%

**Table 5:** Impact of stress of varying Cu^2+^ concentrations (mg/L) on *D. tertiolecta* cells’ chlorophyll content (µg/ml) during in the presence and absence of phosphorus in culturing medium.

Days	Chlorophylls	Normal culture	Culture (zero phosphorus)	Chlorophyll content according to metal concentration				
				5 mg/L	10 mg/L	15 mg/L	20 mg/L	25 mg/L
4	Chlorophyll “a”	0.65	0.62	0.64	0.52	0.40	0.30	0.22
	Chlorophyll “b”	0.21	0.20	0.19	0.16	0.14	0.11	0.09
	Chlorophyll “a + b”	0.86	0.82	0.83	0.68	0.54	0.41	0.29
	Chlorophyll “a/b” ratio	3.1:1	3.1:1	3.4:1	3.3:1	2.9:1	2.7:1	2.3:1
8	Chlorophyll “a”	1.18	1.16	1.20	0.79	0.41	0.31	0.20
	Chlorophyll “b”	0.41	0.41	0.46	0.32	0.22	0.17	0.12
	Chlorophyll “a + b”	1.68	1.57	1.66	1.11	0.63	0.48	0.32
	Chlorophyll “a/b” ratio	2.9:1	2.8:1	2.6:1	2.5:1	1.9:1	1.8:1	1.7:1
12	Chlorophyll “a”	1.52	1.32	1.32	0.84	0.40	0.28	0.16
	Chlorophyll “b”	0.53	0.51	0.50	0.36	0.21	0.17	0.12
	Chlorophyll “a + b”	2.05	1.83	1.82	1.20	0.61	0.45	0.28
	Chlorophyll “a/b” ratio	2.9:1	2.6:1	2.6:1	2.3:1	1.9:1	1.6:1	1.3:1
16	Chlorophyll “a”	1.52	1.36	1.32	0.82	0.22	0.18	0.10
	Chlorophyll “b”	0.64	0.62	0.60	0.54	0.19	0.16	0.09
	Chlorophyll “a + b”	2.16	1.71	1.92	1.36	0.42	0.34	0.19
	Chlorophyll “a/b” ratio	2.4:1	2.2:1	2.2:1	1.5:1	1.1:1	1.1:1	1.1:1

**Table 6:** Percent of decrease in chlorophyll content in *D. tertiolecta* under phosphorus limitation at different concentrations of Cu^2+^.

Day	Element concentration					
	Chlorophyll Type	5 mg/L	10 mg/L	15 mg/L	20 mg/L	25 mg/L
4	Chl a	1.54%	20.00%	38.46%	53.85%	66.15%
	Chl b	4.76%	23.81%	33.33%	47.62%	57.14%
	Chl a + b	4.65%	20.93%	34.88%	52.33%	66.28%
8	Chl a	−1.69%	33.05%	65.25%	73.73%	83.05%
	Chl b	−12.20%	21.95%	46.34%	58.54%	70.73%
	Chl a + b	1.19%	33.93%	62.50%	71.43%	80.95%
12	Chl a	13.16%	44.74%	73.68%	81.58%	89.47%
	Chl b	5.66%	32.08%	60.38%	67.92%	77.36%
	Chl a + b	10.73%	41.46%	70.24%	78.05%	86.34%
16	Chl a	10.53%	46.05%	85.53%	88.16%	93.42%
	Chl b	6.25%	15.63%	70.31%	75.00%	85.94%
	Chl a + b	11.11%	37.04%	80.28%	84.26%	91.20%

### Impact of phosphorus availability on photosynthetic activity (O_2_-evolution calculated as µ mol O_2_ mg chl-1 h-1) of *D. tertiolecta* in presence of different heavy metal concentrations

One of the main physiological processes influencing algal development is algal photosynthetic activity which all aquatic ecosystems’ composition and functioning are impacted. As a result, researching the algal photosynthetic response can be a helpful method for first heavy metal toxicity screening. It should be acknowledged that basic acute ecotoxicity assessments are only a portion of the environmental impact assessment and do not fully capture the effects of solvents released into the environment^56^. Algal physiology is likewise significantly impacted by phosphorus depletion, whether it be through hunger or constraint, albeit with varying changes in photosynthetic activities^57^.

**Fig. 4:**
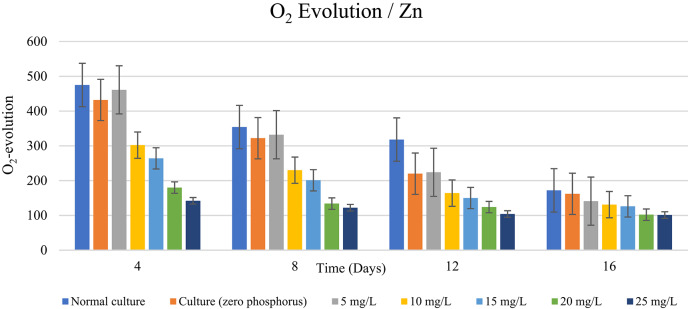
Impact of stress of varying Zn^2+^ concentrations (mg/L) on *D. tertiolecta* cells’ photosynthetic activity (O_2_-evolution calculated as µ mol O_2_ mg chl-1 h-1)) during in the presence and absence of phosphorus in culturing medium.

**Fig. 5:**
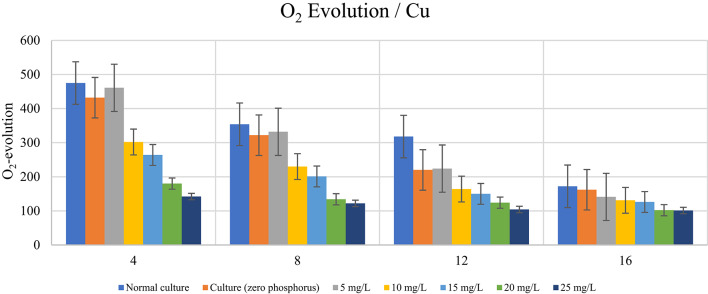
Impact of stress of varying Cu^2+^ concentrations (mg/L) on *D. tertiolecta* cells’ photosynthetic activity (O_2_-evolution calculated as µ mol O_2_ mg chl-1 h-1)) during in the presence and absence of phosphorus in culturing medium.

According to the data in Table 7 and the graphs in Figs. 4 and 5, the phosphorus-starved culture produced less O_2_-evolution until the end of the experiment than the typical phosphorus-containing culture. In comparison to the typical control, the evolution of O_2_ after 4 days of culturing dropped by 8.7%, and after 12 days, it dropped by 30.4%. Phosphorus-starved cells’ O2-evolution rate decreased gradually as a result of varying Zn^2+^ and Cu^2+^ concentrations exceeding 5 mg/L. With the exception of Cu^2+^, which showed a decrease in O_2_-evolution on the sixteenth day of culture, the results of the application of 5 mg/L Zn^2+^ and Cu_2+_ demonstrated that the rate of O_2_-evolution grew under the influence of the two elements examined until the completion of the experiment. The element’s kind and the duration of the culturing period, however, affect the rate of rise in O_2_-evolution. On the eighth day of culture, for instance, the rate of rise in photosynthesis for Zn^2+^ and Cu^2+^ rose by 6.2%, 5.6%, and 3.1%, respectively, in comparison to the phosphorus-starved control. Following a 12-day culture period, the rate of increase in O_2_ evolution for Zn^2+^ and Cu^2+^ was close to 4.5% and 1.8%, respectively, in comparison to the phosphorus-starved control. The data in Table 8 cleared also that Zn^2+^ and Cu^2+^ concentrations over 5 mg/L caused gradual decrease in O_2_-evolution reaching minimum at higher concentrations and at the end of the experiment. Phosphorus-limitation inhibit photosynthetic activity, as evidenced by lower O₂ evolution rates at different metal concentrations. Low Zn²⁺ and Cu²⁺ concentrations had a mild effect on O₂ evolution in normal cultures, but a dramatic reduction was observed above 10 mg/L. Under phosphorus limitation, O₂ evolution decreased significantly, with Cu²⁺ at 25 mg/L generating a 70.11% reduction and Zn²⁺ leading to a 65.05% decline. Significant differences (*p* < 0.001) between phosphorus treatments indicate that phosphorus is necessary for effective electron transport and oxygen evolution in photosynthesis. Cu²⁺ impairs photosynthetic activity more than Zn²⁺, especially in phosphorus-limited environments. This may be due to its higher interference with photosystem integrity and enzyme performance.

In *D. tertiolecta* cultivated for 4 and 12 days under the influence of 10 and 25 mg/L Zn^2+^ and Cu^2+^ ions, the acquired data shows the percentage of decrease in O_2_-evolution. This table makes it evident that the element’s kind, concentration, and culturing time all had a significant impact on the pace of O_2_-evolution. The rate of O_2_-evolution decreases with increasing element concentration and cultured time. Our results are consistent with those of El-Agawany^58^ who demonstrated that heavy metals toxicity reduces the amount of photosynthesis in algae.

Additionally, it was demonstrated by El-Agawany and Kaamoush^14^ that the effect of phosphorus-limiting conditions on the photosynthetic activity of *Dunaliella sp* cells in response to different concentrations of dissolved nickel was less than that of normal cultures (containing phosphorus) in terms of O_2_-evolution.

Our findings are corroborated by Volland et al.^59^ who reported that certain algal enzymes involved in photosynthesis are zinc-dependent and maintained by low concentrations of zinc ions, which promote oxygen evolution in algae. Twiss and Nalewajko^60^ investigated the role of cellular polyphosphate content in mitigating the detrimental impact of copper on photosynthesis in *Scenedesmus acutus* cells. They proposed that polyphosphate serves as a passive defence mechanism, meaning that the higher the cellular P content, the less photosynthesis was inhibited during copper exposure. When *Chlamydomonas reinhardtii* was starved of P, Wykoff et al.^61^ found that the amount of photosynthesis drastically decreased. The light-saturated rate of photosynthesis, as determined by O_2_ evolution, decreased by about 70% following 4 days of P deprivation compared to standard culture conditions. The yield of O_2_ evolution may decrease as a result of a less effective transfer of excitation energy to the PSII process.

As shown in Table 8*Dunaliella tertiolecta*’s photosynthetic oxygen evolution dropped as Zn²⁺ and Cu²⁺ concentrations increased. The determined percentage decreases indicated a clear concentration-dependent suppression of photosynthetic activity. At day 4, Zn²⁺ exposure resulted in decreases ranging from 0.84% at 5 mg/L to 65.05% at 25 mg/L. Cu²⁺ induced higher inhibition, reaching 70.11% at the highest dosage. Similar inhibitory patterns were seen at later sample dates, with significant decreases identified on days 8 and 12. Overall, Cu²⁺ inhibited oxygen evolution more than Zn²⁺, especially at concentrations over 15 mg/L, indicating a larger harmful impact of copper on the alga’s photosynthetic system.

**Table 7:** Impact of stress of varying Zn^2+^ and Cu^2+^ concentrations (mg/L) on *D. tertiolecta* cells’ photosynthetic activity (O_2_-evolution calculated as µ mol O_2_ mg chl^[-1]^ h^[-1]^ during in the presence and absence of phosphorus in culturing medium.

Days	Control		Element	O_2_-evolution according to metal concentration (mg/L)					F (p)	LSD
				5	10	15	20	25		
4	Normal culture	475.00±15.72								
	Culture (zero phosphorus)	432.00±13.08^a^	Zn^2+^	471.00±13.87 ^b^	386.00±11.35 ^c^	343.00±11.52 ^d^	241.00±7.95 ^e^	166.00±8.18 ^f^	324.296** (<0.001)	16.321
			Cu^2+^	461.00±11.36 ^b^	302.00±8.72 ^c^	264.00±7.81 ^d^	180.00±7.00 ^e^	142.00±8.89 ^f^	534.895** (<0.001)	14.119
8	Normal culture	354.00±9.54								
	Culture (zero phosphorus)	322.00±7.94 ^a^	Zn^2+^	340.00±10.58 ^b^	256.00±7.94 ^c^	231.00±7.82 ^d^	146.00±7.95 ^e^	130.00±7.81 ^f^	324.739** (<0.001)	12.217
			Cu^2+^	332.00±9.64 ^a^	230.00±7.00 ^b^	201.00±6.25 ^c^	134.00±6.08 ^d^	122.00±5.29 ^d^	469.928** (<0.001)	10.442
12	Normal culture	318.00±7.21								
	Culture (zero phosphorus)	220.00±8.19 ^a^	Zn^2+^	230.00±7.00 ^b^	187.00±7.81 ^c^	182.00±8.66 ^c^	153.00±8.88 ^d^	115.00±7.01 ^e^	208.694** (<0.001)	11.579
			Cu^2+^	224.00±6.25 ^b^	164.00±7.00 ^c^	150.00±7.94 ^d^	124.00±6.25 ^e^	104.00±4.58 ^f^	280.241** (<0.001)	9.904
16	Normal culture	172.00±5.68								
	Culture (zero phosphorus)	162.00±7.81 ^a^	Zn^2+^	162.00±5.57^a^	143.00±6.93 ^b^	132.00±6.25 ^c^	115.00±7.21 ^d^	102.00±3.61 ^e^	44.469** (<0.001)	9.279
			Cu^2+^	141.00±7.21 ^b^	131.00±5.29 ^bc^	126.00±7.21 ^c^	102.00±5.29 ^d^	101.00±2.65 ^d^	43.245** (<0.001)	8.969

*Statistically significant at p ≤ 0.05, **Statistically significant at p ≤ 0.001, F : F-test (ANOVA), Least significance difference at 0.05, Different superscripts are significant.

**Table 8:** Percent of O₂ Evolution Reduction in *D. tertiolecta* under phosphorus limitation at different concentrations of Zn^2+^and Cu^+ 2^.

Days	Element concentration									
	5 mg/L		10 mg/L		15 mg/L		20 mg/L		25 mg/L	
	Zn^2+^	Cu^2+^	Zn^2+^	Cu^2+^	Zn^2+^	Cu^2+^	Zn^2+^	Cu^2+^	Zn^2+^	Cu^2+^
4	0.84%	2.95%	18.74%	36.42%	27.79%	44.42%	49.26%	62.11%	65.05%	70.11%
8	3.95%	6.21%	27.68%	35.03%	34.75%	43.22%	58.76%	62.15%	63.28%	65.54%
12	27.67%	29.56%	41.18%	48.43%	42.77%	52.83%	51.89%	61.01%	63.84%	67.30%
16	5.81%	18.02%	16.86%	23.84%	23.26%	26.74%	33.14%	40.70%	40.70%	41.28%

### Impact of phosphorus availability on photosynthetic activity (O_2_-uptake) of *D. tertiolecta* in presence of different heavy metal concentrations

The suppression of the photosynthetic machinery is one of the many potential targets of heavy metal-induced harm in photosynthetic organisms. Exposure to heavy metals decreased the amount of chlorophyll in several plants. Phosphate limitation in various aquatic environments increased toxicity even more^62^.

**Fig. 6:**
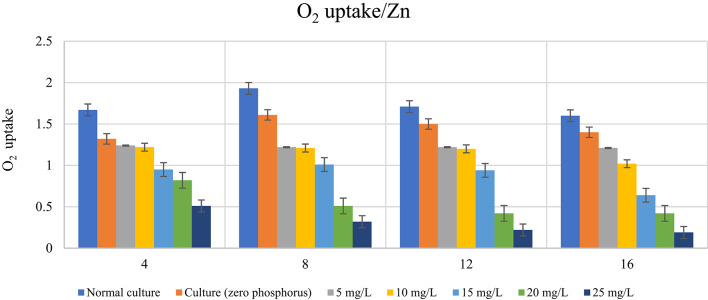
Impact of stress of varying Zn^2+^ concentrations (mg/L) on *D. tertiolecta* cells’ photosynthetic activity (O_2_-uptake calculated as µ mol O_2_ h^− 1^) during in the presence and absence of phosphorus in culturing medium.

**Fig. 7:**
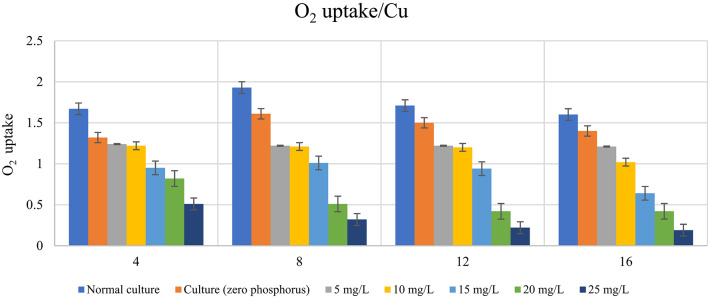
Impact of stress of varying Cu^2+^ concentrations (mg/L) on *D. tertiolecta* cells’ photosynthetic activity (O_2_-uptake calculated as µ mol O_2_ h-1) during in the presence and absence of phosphorus in culturing medium.

The amount of O_2_-uptake by *D. tertiolecta* under phosphorus-starved treated cultures likewise depended mostly on the element’s type and concentration as well as the duration of the culturing time, according to data shown in Table 9 and seen in Figs. 6 and 7. Zn^2+^ was only elevated by 10 mg/L with regard to the untreated phosphorus-starved control, according to the data in this table. Following eight days of culture, the rate of O_2_-uptake for Zn^2+^ rose to 10 mg/L. Between the 12th and 16th days, the rate of respiration only rose at 5 mg/L Zn^2+^ concentration at the other values, it gradually reduced as the concentration of these two elements grew.

Conversely, O_2_-uptake gradually decreased due to Cu^2+^ ions at all concentrations and overall culture times. With increasing Cu^2+^ ion levels and culturing time, the rate of decline progressively increased, reaching its minimum value at 25 mg/L and 16 days into the cultural process. However, for the organism grown for 4, 8, 12, and 16 days, Table 10 displayed the percentage increase or decrease in O_2_-uptake at doses of 5, 10, and 25 mg/L Zn^2+^ and Cu^2+^. The oxygen uptake (respiration) findings supported the negative impact of metal stress in the absence of phosphorus. Zn²⁺ reduced O₂ absorption by 80.00% under phosphorus limitation, while Cu²⁺ reduced it by 88.13% at concentration 25 mg/L. Zn²⁺ only decreased respiration by 25.15% at concentration 15 mg/L, but Cu²⁺ had the same trend by 43.11%. Significant differences (*p* < 0.001) between phosphorus treatments indicate that phosphorus deficiency exacerbates the harmful effects of heavy metals on cellular metabolism. This illustrates how phosphorous protects stressed cells’ respiratory systems. The findings highlight the significance of phosphorus availability in reducing the adverse effects of Zn^2+^ and Cu^2+^, especially in severely contaminated regions.

According to Gao et al.^30^, phosphorus (P) deficit may result in decreased metal detoxification at low cell P quota, which could raise microalgae’s vulnerability to harmful trace metals. They investigated the growth and bioaccumulation of Zn and P in the green microalgae *Pseudokirchneriella subcapitata* and discovered that a lack of P can increase metal toxicity. For instance, by reducing the initially used P content from 186 µg/L to 18.6 µg/L, Cu toxicity to *P. subcapitata* quadrupled in batch cultures with other nutrients at the same initial concentrations as in medium. Additionally, Andresen et al.^62^ investigated the relationship between nickel toxicity and phosphorus limitation in certain aquatic plants and discovered that while metals had a greater impact on the chlorophyll fluorescence of leaves in low P solutions than in high P solutions, P had a greater impact on various physiological parameters (such as oxygen fluxes and chlorophyll content) than did metals. Moreover, according to El-Agawany and Kaamoush^9^, *Dunaliella sp.* respiration rates were higher than those of untreated phosphorus-starved (control) over a short length of time (one week) when 5 mg/L of dissolved nickel was present. At higher concentrations, however, the rate of respiration decreased gradually until the sixteenth day. According to some other studies that corroborated our findings, *Nitzschia closterium* growth rate was cut in half by 20 µg/L of Cu, while photosynthesis was unaffected until copper concentrations above 100 µg/L. Copper’s effects on algal respiration vary depending on the length of exposure, ranging from suppression in *Chlorella sorokiniana* and *Amphidinium carterae* to increase in *Nitzschia closterium* and *Chlorella vulgaris*^63^. Copper was found to be the highest toxic metal against growth and all metabolic parameters compared to zinc and nickel in *Dunaliella salina* alga used as biological marker in detecting pollution in saline lakes^64^.

**Table 9:** Impact of stress of varying Zn^2+^ and Cu^2+^ concentrations (mg/L) on *D. tertiolecta* cells’ photosynthetic activity (O_2_-uptake calculated as µ mol O_2_ h^− 1^) during in the presence and absence of phosphorus in culturing medium.

Days	Control		Element	O2-uptake according to metal concentration (mg/L)					F (p)	LSD
				5	10	15	20	25		
4	Normal culture	^1.67±0.11^								
	Culture (zero phosphorus)	^1.32±0.08 a^	Zn^2+^	1.45±0.11 a	1.42±0.08 a	1.25±0.09 b	1.03±0.08 c	0.55±0.05 d	47.232** (<0.001)	0.125
			Cu^2+^	1.24±0.10 a	1.22±0.10 a	0.95±0.05 b	0.82±0.06 b	0.51±0.07 c	45.381** (<0.001)	0.116
8	Normal culture	^1.93±0.05^								
	Culture (zero phosphorus)	^1.61±0.15b^	Zn^2+^	1.65±0.12 a	1.65±0.15 a	1.21±0.07 b	0.55±0.03 c	0.52±0.08 c	70.705** (<0.001)	0.162
			Cu^2+^	1.22±0.08 b	1.21±0.11 b	1.01±0.06 c	0.51±0.05 d	0.32±0.03 e	95.956** (<0.001)	0.124
12	Normal culture	^1.71±0.10^								
	Culture (zero phosphorus)	^1.50±0.14 a^	Zn^2+^	1.51±0.15 a	1.35±0.15 a	1.01±0.09 b	0.51±0.06 c	0.34±0.03 c	40.390** (<0.001)	0.202
			Cu^2+^	1.22±0.07 b	1.20±0.06 b	0.94±0.08 c	0.42±0.03 d	0.22±0.04 e	59.449** (<0.001)	0.163
16	Normal culture	^1.60±0.12^								
	Culture (zero phosphorus)	^1.40±0.13 a^	Zn^2+^	1.40±0.11 a	1.32±0.05 a	0.96±0.08 b	0.42±0.06 c	0.32±0.07 c	47.183** (<0.001)	0.147
			Cu^2+^	1.21±0.08 b	1.02±0.03 c	0.64±0.06 d	0.42±0.05 e	0.19±0.03 f	85.417** (<0.001)	0.129

*Statistically significant at p ≤ 0.05, **Statistically significant at p ≤ 0.001, F : F-test (ANOVA), Least significance difference at 0.05, Different superscripts are significant.

**Table 10:** Percent of O_2_-uptake Reduction in *D. tertiolecta* under phosphorus limitation at different concentrations of Zn^2+^and Cu^+ 2^.

Days	Element concentration									
	5 mg/L		10 mg/L		15 mg/L		20 mg/L		25 mg/L	
	Zn^2+^	Cu^2+^	Zn^2+^	Cu^2+^	Zn^2+^	Cu^2+^	Zn^2+^	Cu^2+^	Zn^2+^	Cu^2+^
4	13.17%	25.75%	14.97%	26.95%	25.15%	43.11%	38.32%	50.90%	67.06%	69.46%
8	14.51%	36.27%	14.51%	37.31%	37.31%	47.67%	71.50%	73.58%	73.06%	83.42%
12	11.70%	28.65%	21.05%	29.82%	40.94%	45.03%	70.18%	75.44%	80.12%	87.13%
16	12.50%	24.37%	17.50%	36.25%	40.00%	60.00%	73.75%	73.75%	80.00%	88.13%

The study fills that gap by offering quantitative proof that metal toxicity is significantly increased by phosphorus restriction. This finding has obvious ramifications for ecological risk assessment and remediation measures in waterways with an imbalance in nutrients, Table 11.

**Table 11:** Summary of recent literatures on algal responses to heavy metal stress under varying nutrient conditions, compared with the current study.

Species	Metal type	Study focus	Key findings (relevance to current study)	Ref. #
Microalgal–bacterial symbiosis (wastewater)	Zinc metals in wastewater	Role of phosphate in treating wastewater containing heavy metals (Zn^2+^)	Phosphate availability is shown to have a significant impact on metal removal and system performance, underscoring the significance of P in reducing metal stress in treatment settings (supports nutrient–metal interaction study).	^6^
*Chlorella vulgaris*, *Scenedesmus obliquus*	Cu, Fe (used for removal tests in aqueous medium)	Metals and nutrient removal performance (phosphate & nitrate; toxicity monitoring)	Our interest in coupled nutrient–metal dynamics is supported by evidence that metal toxicity rises with metal concentration and influences nutrient absorption (P).	^43^
*Thalassiosira weissflogii* (marine diatom)	Zn (various experimental concentrations)	Effects of phosphorus and Zn stress on growth & physiology	Similar to our observation that P availability influences Zn effects, it has been shown that P chemistry alters Zn toxicity and absorption.	^10^
*Pseudokirchneriella subcapitata*	Zn (various concentrations)	Influence of phosphate on Zn toxicity	Observed Phosphate-limiting growth in that system reduces zinc toxicity, indicating that P-metal interactions may vary depending on the species and circumstances. This supports the need for species-specific testing, which is what we conducted.	^30^
*Chlorella vulgaris*	Cd with differing P levels	P controls growth & biochemical composition under Cd stress	Established another cross-study precedent for nutrient–metal interactions by demonstrating that P availability alters Cd toxicity.	^49^
Coastal benthic systems (field study)	Environmental metal levels (spatial/seasonal variation)	Interaction of eutrophication and sediment heavy metal pollution	Highlights the ecological significance of combining P restriction with metal exposure by demonstrating the true co-occurrence of nutrients and metals in the field.	^22^
*Raphidocelis subcapitata*	Al + P-limitation	Phosphorus limitation combined with aluminum exposure	Reported synergistic negative effects of P-limitation and metal stress — a mechanistic precedent for our P and metal interaction approach.	^24^
*Chlamydomonas reinhardtii* and *Scenedesmus obliquus*	Arsenate (various) under different phosphate regimes	Toxicity and bioaccumulation of arsenate under different P regimes	Methodologically relevant background: Metal/metalloid toxicity and accumulation are influenced by the shown phosphate regime, which confirms our results.	^50^
*Dunaliella salina*	Cu, Zn (various experimental concentrations)	Toxicological impacts on growth, amino acids	The discovery of more Cu toxicity than Zn on growth and amino acids supports our conclusion that Cu is more toxic than Zn.	^11^
*Dunaliella tertiolecta*	Ni, Cu, Zn	Effect of different heavy metal concentrations on protein content	Showed significant protein decreases (Cu strongest) — supports measured biochemical stress endpoints in our study.	^16^
*Dunaliella* sp. AL-1	Cr and Cu in aqueous solutions	Biochemical & physiological responses to Cr and Cu	Recent findings support our Cu-dominant toxicity pattern by demonstrating increased Cu toxicity and changed pigments and physiology.	^17^

## Conclusion

This study highlights the critical role of phosphorus availability significantly influences the response of *Dunaliella tertiolecta* to heavy metal stress, with phosphorus limitation exacerbating the toxic effects of Cu²⁺ and Zn²⁺. The EC50 values for both metals were around 15 mg/L, indicating moderate toxicity. Cu²⁺ has more toxicity than Zn²⁺, resulting in decreased growth, chlorophyll content, and photosynthetic activity.

In contrast to phosphorus-starved cultures, algal growth was markedly suppressed under phosphorus-starved conditions, and the rate of increase in optical density was slower. In the absence of phosphorus, Zn²⁺ initially stimulated chlorophyll formation more effectively than Cu²⁺; however, at higher doses (20–25 mg/L), both metals caused a reduction in chlorophyll synthesis. Nevertheless at all tested concentrations, Cu^2+^ inhibited chlorophyll production more significantly than Zn^2+^.

Phosphorus limitation dramatically decreased photosynthetic activity, as indicated by O₂ evolution, especially at metal concentrations greater than 10 mg/L. In contrast to the phosphorus-starved control, photosynthesis briefly increased at lower metal concentrations (5 mg/L), indicating a potential stimulatory impact prior to poisoning. Cu^2+^ had a more noticeable effect than Zn^2+^, and O₂ absorption steadily declined as metal concentrations rose, suggesting metabolic inhibition.

These results highlight the need of taking into account both metal contamination and phosphorus availability when evaluating environmental concerns, and they offer fresh insights into how nutrient constraint affects metal toxicity in aquatic systems. The study also highlights *D. tertiolecta’s* potential for bioremediation applications since nutritional conditions affect the organism’s capacity to withstand and reduce metal stress. In order to better understand the ecological and biotechnological relevance of these interactions, future studies should concentrate on intracellular metal buildup, molecular stress responses, and long-term adaption mechanisms.

###  Recommendations

1. Improving the Management of Nutrients in Polluted Waters. When evaluating the risks of heavy metal pollution in aquatic ecosystems, adequate nutritional balance should be taken into account because phosphorus availability affects metal toxicity.
2. To more accurately forecast ecological effects on aquatic microorganisms, environmental monitoring strategies should incorporate both nutrient levels and heavy metal concentrations.
3. Regulation of Heavy Metals in Industrial Effluents. These industries should use integrated treatment systems that address both metal toxicity and nutrient imbalances.

### Future research directions

- Toxicity Mechanisms: Examine the precise mechanisms of heavy metal toxicity in *D. tertiolecta*, such as metal binding to cellular constituents, oxidative stress, and metabolic pathway disruption.
- Bioremediation Possibility: Examine *D. tertiolecta*’s capacity to bioremediate waters contaminated by heavy metals, especially in various nutritional environments.
- Productive Effects: Examine how many stressors, including nutrient constraint, heavy metals, and other environmental conditions, might affect algal communities in concert.
- Research investigations: To determine how nutrient limitation and heavy metal contamination affect natural algal populations in pertinent aquatic environments, conduct field investigations.

The research findings discuss some ecological effects of nutrient constraint and heavy metal contamination in aquatic ecosystems can be better understood in light of these discoveries. The complex interactions between these stressors and their effects on algal communities and ecosystem function require more investigation.

## Supplementary Information

Below is the link to the electronic supplementary material.

Supplementary Material 1

## Data Availability

All data generated or analyzed during this study are included and available in this manuscript.
