# Supplementary material for: Nutrient metal interactions and adaptive responses of Dunaliella tertiolecta to zinc and copper toxicity under phosphorus limitation
Source: Sci Rep. 2026 Apr 24;16:13399. doi: 10.1038/s41598-026-47929-1 (PMC13109415; doi:10.1038/s41598-026-47929-1)
Supplement: Supplementary file 1 — Supplementary Material 1 [file 41598_2026_47929_MOESM1_ESM.docx]

**Environmental implication**

These results highlight how crucial it is to comprehend how many stressors interact to affect aquatic ecosystems. We can aim to safeguard the integrity and well-being of our water resources by tackling the problems of nutrient enrichment and heavy metal pollution.

**1. Aquatic Ecosystem Disruption:**

- **Decreased Primary Productivity:** Algal growth and photosynthesis are greatly impacted by phosphorus constraint and heavy metal contamination. Higher trophic levels, such as fish and other creatures, may be impacted by this decreased primary productivity, which has the potential to upset the entire aquatic food chain.
- **Biodiversity Changes:** In aquatic environments, nutrient constraints and heavy metal stress-induced algae population declines can result in a reduction in biodiversity. The general stability and well-being of the ecosystem may be negatively impacted by this loss of biodiversity.

**2. Deterioration of Water Quality:**

- **Increased Respiration rate:** The study explained that algae suffering from heavy metal stress changed the oxygen rate inside the cell. This higher oxygen use might worsen oxygen depletion in water bodies, especially in areas with poor water circulation and high pollution levels.
- **Heavy Metal Accumulation:** Algal biomass has the potential to exhibit heavy metal accumulation. The consumption of these algae by other organisms may cause heavy metals to bioaccumulate in the food chain, endangering human health.

**3man Health consequences:**

- **Seafood quality:** Consuming heavy metal-contaminated seafood might offer serious health concerns.
- **Heavy metal contamination:** in water sources can have an influence on human health, including drinking water.

**4. Adverse effects on economy:**

**• Declining Fish Populations:** Fisheries and other sectors may experience substantial financial effects as a result of the decrease food availability.

**5. Future Strategies:**

**• Pollution Control:** To lessen the amount of heavy metal and nutrient runoff into water bodies, stronger rules on agricultural and industrial discharges should be put in place.

**• Sustainable Agriculture:** Encouraging environmentally friendly farming methods that reduce the need for pesticides and fertilizers.

**• Wastewater Treatment:** Enhancing wastewater treatment methods “ especially environmental friendly methods” to eliminate nutrients and heavy metals prior to release.
